# Research Ethics Committees (RECs) and epidemic response in low and middle income countries

**DOI:** 10.11604/pamj.2018.31.209.17076

**Published:** 2018-11-28

**Authors:** Luchuo Engelbert Bain, Chia Gerald Ngwain, Julius Nwobegahay, Jeffery Gabriel Sumboh, Rogers Nditanchou, Paschal Kum Awah

**Affiliations:** 1 Athena Institute for Research on Innovation and Communication in Health and Life Sciences, Vrije Universiteit Amsterdam; 2 Centre for Population Studies and Health Promotion, CPSHP, Yaounde , Cameroon. BP 7535, Yaounde; 3 Northwest Hospital, 5401 Old Court Road, Randallstown, MD, 21133; 4 Military Health Research Centre (CRESAR), Yaounde, Cameroon; 5 Yaounde Military Hospital, Yaounde, Cameroon; 6 Department of Parasitology, Noguchi Memorial Institute for Medical Research, University of Ghana. P.O Box 581. Legon; 7 Regional Coordination, National Programme for the Fight Against Tuberculosis, North Region, Cameroon; 8 Department of Anthropology, Faculty of Arts, Letters and Social Sciences, FALSS, University of Yaounde I, Cameroon

**Keywords:** Luchuo Engelbert Bain, Athena Institute for Research on Innovation and Communication in Health and Life Sciences, Vrije Universiteit Amsterdam

## Abstract

The recent Ebola and Zika virus epidemics in some parts of Africa and Asia have showcased the porosity in disaster preparedness and response, not only in the affected countries, but on a global scale. For the Ebola epidemic, scientifically robust research was started late during the course of the epidemic, with waste of resources and lost research opportunities. Research Ethics Committees have a significant role to play with regards to epidemic response for the future. This paper presents key challenges and opportunities for ethics review during emergencies, specifically for low and middle income countries. There is no better moment to test the efficacy and safety of drugs or vaccines for infected, or at risk populations than during the disaster itself. The main mantras that form the back bone of research ethics review (Helsinki Declaration, the CIOMS International Ethical Guidelines for Biomedical Research Involving Human Subjects, WHO and the ICH guidelines for Good Clinical Practice) are increasingly showing their limitations. Most protocols are generally from developed countries where the funding originates. Not only is the direct transposition to Low and Middle Income Country (LMIC) settings inappropriate on its own, also, using such guidelines in times of public health disasters might be time consuming, and might also lead to wastage of research opportunities, especially when sociocultural peculiarities, and anthropological research arms are completely excluded or avoided within the care and research packages. Governments should include RECs as key members during the elaboration, and daily functioning of their national public emergency response packages. Developing simple research ethics review guidelines, involvement of health care staff in ethics training, community mobilization, and incorporation of anthropological research during the medical response, research and communication phases, are imperatives in epidemic response.

## Introduction

The recent Ebola and Zika virus epidemics in some parts of Africa and Asia have showcased the porosity in disaster preparedness and response, not only in the affected countries, but on a global scale [1, 2]. The response of the international community, especially the World Health Organization, humanitarian organizations like Medecins Sans Frontiers (Doctors Without Borders (MSF) and other research partners has been described as late and exemplified by huge collaboration and coordination loopholes. For instance, it took the WHO over 8 months to declare the Ebola epidemic as a global public health concern [2], and well scientifically robust clinical trials came into the scene at the end of the crisis. Obtaining ethical approval from local ethics committees, as well as international collaborating partners for research protocols was a serious hindrance for timely initiation of the research process [2-4]. Inexperience and inadequate expertise for rapid and high quality review in emergencies, as well as weak collaboration and coordination gaps in cross-country /institutional research endeavors played significant roles for the late start of these studies [2, 5, 6].

Research during these disasters provides appreciation of the gravity of such disasters, maps disease spread and key drivers, guides response, permits monitoring and evaluation of interventions, and allows for a keener look at the natural evolution of disease and the rate of mortality and morbidity [2,3]. In conditions with unknown or unproven causes of diseases or available interventions, ethics review may be compelled to shift from the slow and traditional research ethics review process to a more rapid and properly guided approach; for instance, risk—benefit analysis, informed consent and vulnerability [1, 3]. Transdisciplinary research teams, though difficult to constitute and coordinate, remain useful in properly handling public health emergency responses, from acute case identification and management, to research and proper anthropological appraisal of specificities of the affected regions. This does not only permit proper and appropriate recommended health practices, but could also go further to enhance trust existing between the response teams and the community. Carrying out research, alongside emergency and humanitarian response, is an ethical obligation during public health disasters [5, 7]. Research Ethics Committees (RECs) in low and medium income countries need to be prepared, to properly review protocols in public health disasters in a timely manner, without losing protocol review quality. There is no better moment to test the efficacy and safety of drugs or vaccines for infected or at-risk human beings than during the public health disaster itself [2]. In the case of the recent Ebola epidemic, over nine clinical trials were carried out when the epidemic had almost disappeared. It is regrettable that most scientifically rigorous clinical research efforts were initiated too late to yield useful data [1, 2, 4, 6].

It is elusive and idle to contend that ethics review during disease can adhere to specific guidelines. The same disaster in different regions of the world mandate different considerations in the ethics review process (health system, human resource, sociocultural peculiarities). It is increasingly very common to accept deviations from standard review guidelines in disasters [8]. This, in no way, should put the ethical backbone of humanitarian, medical and research obligations of the respective intervening teams into the shadows [8]. The vulnerability of the affected countries and peoples is a reality during disasters, and deserves to be addressed with a lot of caution [9]. A poor assessment could lead to the exploitation of research participants, potential lack of trust or unnecessary exclusion of certain groups of persons that deserve to be researched upon, depending on the situation (e.g pregnant women as with the Zika virus epidemic and children). On the other hand, a poor demarcation of the concept of vulnerability can exclude specific groups from benefiting from research (pregnant women and children). For instance, mortality in Ebola was high as early as from the 2nd to 3rd day after contamination. This contrasts with the lengthy timeframes from submission of research protocols, to obtaining approval from RECs. In a review, reported by Rishu *et al.* [10], these could range from 42 to 188 days during the Zika and Ebola pandemics. We attempt to highlight a few areas to be considered in developing ethics review frameworks, as well as reviewing research protocols during emergencies in LMICs. How can LMICs be prepared to accelerate ethics review in public health emergencies while upholding high ethical standards, is the central question that we attempt to answer in this essay.

## Discussion

### Preparing Research Ethics Committees in Low and Medium Income Countries (LMICs) for the next Pandemic

Well prepared RECs for rapid protocol review remain ethical and scientific priorities, to better control and coordinate future public health disasters. Strengthening of Research Ethics Committees competence in rapid ethics review is a key recommendation that requires immediate action before the next pandemic [4-6]. Clinical research during emergencies remains a key research opportunity not to be missed. Coordination, control and collaboration frameworks of the RECs need to be carefully reconsidered, more than ever before, since an epidemic somewhere remains a risk everywhere [1, 7-8]. The obstacles to conducting emergency research in LMICs include: obtaining timely research clearances, forging new collaborations, training research staff, negotiating material transfer agreements, sourcing and importing drugs and sophisticated equipments, and ensuring access to a reliable power [1-3]. It is a moral and ethical obligation for countries and the international scientific community to prepare RECs for rapid ethics review before the next pandemic. This is particularly concerning for RECs in LMIC, which are generally underfunded, have members without formal expertize in research ethics and receive very little support from the respective states. Funding and weak institutional support still characterize most of the RECS in LMICs [9, 11]. The will of the governments of LMICs to make enabling legislation available is the most intriguing situation, as most of them lack or have weak research regulations.

The main mantras that form the back bone of ethics review (Helsinki Declaration, the CIOMS International Ethical Guidelines for Biomedical Research Involving Human Subjects, WHO and the ICH guidelines for Good Clinical Practice) are increasingly showing their limitations [1,2, 4]. Not only is the appropriateness of direct transposition to LMIC settings inappropriate on its own [12], using such guidelines in times of public health disasters might also be time consuming and lead to waste of research opportunities, especially when sociocultural peculiarities and anthropological research arms are completely excluded or avoided within the research package [2, 12]. Strengthening and building capacity of REC members in most LMICs remain an urgent imperative. Most of them receive little or no support from respective governments, have members not properly trained in research ethics, and operate on altruistic grounds [11]. This could predispose them to unconsciously underappreciate the ethical appropriateness of research protocols from elsewhere. This becomes even more concerning in cases of emergencies. Richardson *et al.* have advocated support of local RECs during emergencies to provide robust and rapid ethical review [8]. Without underrating the importance of such support, preparing country specific review guidelines for public health emergencies upfront would be more productive.

People experiencing a public health emergency, especially with high mortality and morbidity rates, and at times with unknown causes evidence-based treatment options are inherently vulnerable. Doing research in such a situation of uncertainty is a moral mandate for the scientific community. Imagine no research is done during epidemics, then, this will simply mean leaving the world at the expense of future pandemics, since the same inputs, are bound to produce the same outcomes, everything being equal. This however, is no excuse to exert extra unjustified exploitation of these vulnerable populations. Researchers, humanitarian workers and health care providers might unconsciously impose an extra burden or exploit a vulnerable population unknowingly. Training Research Ethics Committee members around the contours of vulnerability assessment in LIMCs is recommended. With a growing amount of work in the ethics literature on vulnerability, frameworks specifically presenting how to assess and deal with vulnerabilities in public health emergencies are sparse. For instance, in the case of Zika virus with microcephaly increasingly being recognized as a complication in pregnant women, should the latter be included in research or trials or not, and under what circumstances, are issues that need a deep ethical and philosophical reflection, which as of now remain lacking. Leaving ethical evaluations exclusively in the hands of bioethicists is misleading. Evidence-based practice on specific subjects could be provided by health care staff or other specialists in emergency response. Not getting such input can distort the ethics evaluation process.The Bioethicists, or REC members would not always have the needed expertise in all fields or contexts. Intellectual and moral humility justify consultation or invitation of experts in such circumstances when needed. It is an overwhelming imperative for healthcare staff in disaster situations to have an idea of potential ethical issues. Ethically responsive medical care is a precondition for successful research in disasters, since healthcare and research often go hand in hand in such circumstances. Failure of the health care team to properly ascertain ethical issues can have far reaching consequences that could hinder the research process. For instance, if the community realizes that they are exploited (sample collection under the canopy of broad consent) without their consent, lack of trust could hinder them from accepting to participate in the research process. These uncertainties could be avoided through basic research ethics training courses (sessions) among health care staff [13].

### Ethics Review, communication and community engagement in public health emergencies

The imperative to speed up ethics review in emergencies not accompanied by low ethical standards remains challenging. RECs in LMIC could play significant roles during public health disasters through provision of timely review, taking into account realities of the local contexts. In emergencies with high mortality and mortality rates, as was showcased by the Ebola crisis, the neglect of inclusion of community actors and considerations during the interventions and studies, can lead to high refusal rates of evidence-based recommendations from health care staff, as well as high attrition rates for most of the clinical trial participants [14]. The community needs to understand the relevance of carrying out research alongside medical care during the disasters [14-16]. Behavioral and communication theories during disaster response specific to Low and Middle Income Countries [LMIC] are almost non-existent. The care and research gaps noticed during the Haiti Cholera outbreaks and the recent deadly Ebola crisis depict the fact that much has not been learned, nor proposed to improve community engagement in these settings. Byron and Howard have exemplified how the strength of appropriate communication techniques, when it comes to making pregnancy decisions in Zika virus, affected countries [17]. A research ethics committee is important to categorize the level of risk with regards to the characteristics of the women in question. For instance, the advice given to a 21 year old with no adverse/contributive obstetric history cannot be similar to that offered to a 41 year lady, with a past history of documented infertility in a Zika affected area. Proper communication strategies do not only enhance trust, but they also offer give room for research participants to make ‘informed choices’, though being within a context of high vulnerability.

The paradigms to undertake rapid anthropological research during disasters could be difficult to set up and be coordinated within a relatively short lapse of time. However, it is a consideration the warrants the attention of the RECs, the response team and researchers. For research during emergencies to be fruitful, close monitoring of community response and attitudes towards the research intervention (For instance, a randomized clinical trial) is a priority. Insensitivity towards community perceptions of the intervention is counterproductive on three grounds. Firstly, distrust in health systems and health care providers with possible non-compliance to recommended intervention under study [18]. Secondly, high attrition rates from trials, and thirdly, return to traditional medical practices, which might be ineffective or even potentially dangerous. It must be remembered that many patients in LMIC do take concomitantly, western medicine and traditional medicine. Ethnographic inputs from Anthropologists and Sociologists are unavoidable and they justify the ethics review process in emergencies to be better appreciated, not as a one point event, but as an ongoing process. Ejeta *et al.* have highlighted the utmost importance of developing behavioral theories and models addressing preparedness needs specific to LMIC [14]. The traditional, The Health Belief Model (HBM), Extended Parallel Process Model (EPPM), Theory of Planned Behavior (TPB) and Social Cognitive Theories have been applied differently in different settings with different outcomes. A scholarly reflection on the strengths and weaknesses of these theories could provide a guide for the pandemic preparedness response plans, which no one knows when it is to come. Regular feedback from the field to inform the ethics committee and research team are necessary to enhance trust, modify communication, and intervention implementation techniques based on specific cases.

Some countries do have existing national emergency response plans. However, whether they do recognize that research during emergencies is a priority component of the response package deserves proper evaluation. Proper education of response team members on the importance of ethics review is needed, as well as inclusion of this review team within the national emergency response strategy.

### Adapting useful requirements for RECs during emergencies

A National Academy of Sciences, Engineering and Medicine expert committee (United States of America) have identified seven moral requirements to be respected during epidemics, which could be useful to RECs [5]: scientific and social value, respect for persons, community engagement, concern for participant welfare and interests, a favorable risk-benefit balance, justice in the distribution of risks and benefits and post-trial access.

It is a moral responsibility on the part of the research team to make affected communities to come to the realization that carrying out research in disaster situations is very vital. They should equally ensure Community engagement at all phases of the emergency response process. A proper communication package though mandatory, to meet its intended goals, mandates community engagement. The presence of members from the community in the response team will not only enhance trust between the researchers and research participants, but will also facilitate rapid anthropological studies needed to guide the health communication packages and identifying socio-culturally sensitive issues to be considered during the research protocol development and implementation phases. It is only through a frank exchange that the research participants will come to appreciate the scientific value of doing research during emergencies. Post trial access remains an important component of clinical trials. Poor relationships between researchers and participants would render this phase of the research process almost impossible. This does not only mean exploitation of participants, wastage of resources with trials generally very expensive, but also, jeopardizing future research endeavors in affected communities.

A proper risk-benefit analysis and the presence of clinical equipoise, especially for clinical trials might not be so easy to evaluate or justify, especially in cases where REC members are inexperienced as was seen during the Ebola outbreak [5]. Countries need to set up working groups and come up with country guidelines on how ethics review needs to be carried out during public health emergencies, and which REC has the competence to do what. This document should have clear definitions of public health emergencies, and which competent REC has the competence to undertake such reviews. It is abominable and just unacceptable for public health disasters to be declared not by country public health officials, but by international bodies. Though cross-country collaboration remains an ideal, health systems strengthening to boost competence of national health systems to be capable of declaring, reacting to and managing these disasters, can only be better undertaken and coordinated by local actors. Clear rules for full and expedited review might be helpful for the organization of such reviews [1, 4, 6]. Adapting standardized or traditional ethics review guidelines during emergencies might be time consuming, inappropriate and could lead to sub-optimal quality of ethics review [12]. A simplified emergency response review framework for countries could be helpful. There is a risk of a mismatch in researcher and community perceptions of what makes research ethical, and this could disrupt trust and render efforts counterproductive with both sides tending to lose [12, 18]. Thielman *et al.* have highlighted the importance of involving trusted actors (participants, researchers and community representatives) during the emergency response period. This becomes very important in research contexts, when participants might need to be followed up for in specific cohort studies [1]. Community engagement could favor ownership of the research by the community, since they would understand potential benefits and goals of the research. Awah *et al.* (2015), have identified distrust and conflict between two healing systems (traditional or local and the Western systems), even amidst life threatening conditions like Ebola [16]. The importance of including anthropological research within the research and emergency response packages during disasters can therefore not be overemphasized. Failure to engage such parties could predispose to premature closure of certain trials.

The document should be preferably made simple for understanding and rapid review. Elsewhere, simplified documents did not reduce the quality of the review process [19]. With a Plethora of RECs in most countries today, usually of questionable ability to properly undertake ethical review, it is not idle to suspect that some researchers would opt for low standard review RECs. Capacity strengthening, community engagement and international coordination/collaboration have been identified as key priorities for engagement in the clinical research agenda in public health disaster response [1, 2, 11].

The RECs have to be flexible to involve experts in specific fields of interest in the review process, though they might not be constitutive members of the REC. Proper evaluation of risk—benefits might need expertise that might be outside the very constitution of the very research ethics committee (neurologists, cardiologists, ophthalmology), depending on the research question under review. Provision of documents in a simple language has been recommended elsewhere [20]. Pretesting of these documents could be helpful. This could be an opportunity, to, in a faster way, ascertain grey areas with the understanding of the meaning, causes, treatment and implications of these epidemics in specific areas.

The appropriateness of the research design is a key scientific and ethical consideration. Sound science is an ethical cornerstone [5]. With most of these studies generally very expensive to carry out (especially under disaster circumstances), it is further unethical to abuse the altruism of research participants for weak or poor quality research, where the findings would not be helpful.

## Conclusion

Collaborative frameworks between research (ers) institutions across countries (funding countries and where research is carried out) are still poorly established, especially regarding management and recognition of ethics approvals. Delays in obtaining ethical approvals in emergencies lead to loss of meaningful research opportunities. Inclusion of anthropological research within the clinical research package, though challenging, constitutes an unavoidable cornerstone within the emergency response package. Governments should include Ethics Review Committee members as key members during the elaboration and daily functioning of their national public emergency response packages. However, the starting point still remains proper training of ethics committee members in research ethics, as well as providing an appropriate climate and funding for these RECs to properly exercise their duties.

## Competing interests

The authors declare no competing interests.
